# Optimization and Validation of Dosage Regimen for Ceftiofur against *Pasteurella multocida* in Swine by Physiological Based Pharmacokinetic–Pharmacodynamic Model

**DOI:** 10.3390/ijms23073722

**Published:** 2022-03-28

**Authors:** Kun Mi, Shanju Pu, Yixuan Hou, Lei Sun, Kaixiang Zhou, Wenjin Ma, Xiangyue Xu, Meixia Huo, Zhenli Liu, Changqing Xie, Wei Qu, Lingli Huang

**Affiliations:** 1 National Reference Laboratory of Veterinary Drug Residues (HZAU) and MAO Key Laboratory for Detection of Veterinary Drug Residues, Huazhong Agricultural University, Wuhan 430070, China; mikun@webmail.hzau.edu.cn (K.M.); hyx97@webmail.hzau.edu.cn (Y.H.); sunlei23@webmail.hzau.edu.cn (L.S.); mawenjin@webmail.hzau.edu.cn (W.M.); HuoMeixia@webmail.hzau.edu.cn (M.H.); liuzhli009@mail.hzau.edu.cn (Z.L.); xiechangqing@mail.hzau.edu.cn (C.X.); 2 MAO Laboratory for Risk Assessment of Quality and Safety of Livestock and Poultry Products, Huazhong Agricultural University, Wuhan 430070, China; pushanju@126.com (S.P.); flyingkai@wemail.hzau.edu.cn (K.Z.); xuxiangyue@webmail.hzau.edu.cn (X.X.)

**Keywords:** ceftiofur, *Pasteurella multocida*, PBPK/PD model, dosage regimen, mathematical model

## Abstract

Model informed drug development is a valuable tool for drug development and clinical application due to its ability to integrate variability and uncertainty of data. This study aimed to determine an optimal dosage of ceftiofur against *P. multocida* by ex vivo pharmacokinetic/pharmacodynamic (PK/PD) model and validate the dosage regimens by Physiological based Pharmacokinetic-Pharmacodynamic (PBPK/PD) model. The pharmacokinetic profiles of ceftiofur both in plasma and bronchoalveolar lavage fluid (BALF) are determined. PD performance of ceftiofur against *P. multocida* was investigated. By establishing PK/PD model, PK/PD parameters and doses were determined. PBPK model and PBPK/PD model were developed to validate the dosage efficacy. The PK/PD parameters, AUC_0–24 h_/MIC, for bacteriostatic action, bactericidal action and elimination were determined as 44.02, 89.40, and 119.90 h and the corresponding dosages were determined as 0.22, 0.46, and 0.64 mg/kg, respectively. AUC_24 h_/MIC and AUC _72 h_/MIC are simulated by PBPK model, compared with the PK/PD parameters, the therapeutic effect can reach probability of target attainment (PTA) of 90%. The time-courses of bacterial growth were predicted by the PBPK/PD model, which indicated the dosage of 0.46 mg/kg body weight could inhibit the bacterial growth and perform good bactericidal effect.

## 1. Introduction

*Pasteurella multocida* (*P. multocida*), a zoonotic gram-negative bacterium, is a causative agent of numeric diseases in human and animals, including pigs, calves, rabbits, and poultry [1]. *P. multocida* is a major pathogen that can lead to respiratory tract disease in pigs, which commonly inhabits mucosal surfaces and upper respiratory and liberates toxins causing necrotic lesions in lung tissue.

Ceftiofur (CEF), a third-generation cephalosporin, is solely approved for veterinary used. It possesses an excellent antibacterial activity against pathogens of swine respiratory tract disease, both in vivo and in vitro [2]. However, as previous report, *Salmonella* Saintpaul was exposure of ceftiofur which increased 2 folds activities of Bla_CTX-M-65_ that leads to decrease the activity of cephalosporin for human used, ceftriaxone. [3]. In addition, antimicrobial resistance gene can transfer to into vegetables [4] or the pathogens that threaten human health [5]. Due to the irrational antibacterial usage, the resistance of *P. multocida* to antibiotic is emerging, and furthermore, producing the interspecies transmission, which seriously threaten to public health [6,7]. Therefore, it is necessary to determine an optimal dosage regimen of CEF against *P. multocida* to guide the clinical medicine and ensure the security in human and animals.

Mathematical models are valuable tools in medicine and public health. The application of pharmacometrics modeling can provide a quantitative description of drug exposures resulting from different dosing regimens as well as insights into their relationships with drug effect [8]. In addition, the US Food and Drug Administration (FDA), European Medicines Agency (EMA), and Central for Drug Evaluation of China encourage to adapt the mathematical model for drug research and development [9]. Pharmacokinetic and pharmacodynamic (PK/PD) model is a valuable tool to determine optimal dosage regimens that can ensure the clinical efficacy on pathogens and alleviate the emergence of resistance [10]. Currently, numerous PK/PD models have been employed in veterinary field and PK/PD indices (fAUC/MIC, fCmax/MIC, and fT > MIC) play an important role [11,12]. By infected target tissue sampling, such as intestinal tract by ultrafiltration probe [13] and lung by bronchoalveolar lavage fluid (BALF) [14], an ex vivo PK/PD model can be established to determine an optimized dosage regime.

However, MIC-based PK/PD parameter is a simplify PK/PD relationship which may ignore the process of PK and PD. The mechanism-based model is developed to predict time-course of effects and drug concentrations. Physiological based pharmacokinetic (PBPK) model, established based on physiological mechanism and drug-physicochemical properties, can be used to predict unbound PK profiles in target tissue [15]. Semi-mechanistic PD model, applying the antibacterial mechanism and drug-resistant mechanism, is employed to elucidate bacterial growth and kill behavior under antibacterial exposure [16]. A PBPK/PD model, integrated by the above models, was exploited for simulation time-course of bacterial growth under antibacterial exposures from target tissues. In addition, the model can capture the emergence and spread of resistance. Currently, some studies have applied them to evaluate the rationality of dose [17,18].

In this investigation, the in vitro PD of ceftiofur against *P. multocida* and PK parameters of ceftiofur in BALF and plasma of *P. multocida*-infected swine were determined. The ex vivo PK/PD model, based on bronchoalveolar lavage technique, was developed and the different target PK/PD indices of ceftiofur against *P. multocida* were simulated. Then, the dosage regimen of ceftiofur against *P. multocida* was proposed and validated by physiological based pharmacokinetic model (PBPK) and semi-mechanistic PD model. The application of PBPK/PD model is essential for determination of dosage regimens that optimize bacterial killing and/or avoid resistance emergence.

## 2. Results

### 2.1. MIC, MBC, MPC and PAE of HB13

*P. multocida* strains (HB13), a wild strain which was isolated from Hubei in 2017, was selected for the PD experiment. The in vitro and ex vivo MIC were both determined as 0.06 µg/mL. The MBC of 0.125 µg/mL for both in vitro and ex vivo. The ratio of MBC/MIC was 2 which signified ceftiofur might have a strong bacteriostatic activity both in vitro and ex vivo. The in vitro MPC were determined as 0.3 µg/mL.

The post-antibiotic effect (PAE) of CEF was determined for 1 × MIC, 2 × MIC, and 4 × MIC by different time exposure (1 h and 2 h). The results were shown in Table 1, PAE was extended along with the increase of co-cultured time and CEF concentration.

### 2.2. In Vitro and Ex Vivo Time-Killing Curves

**Figure 1:**
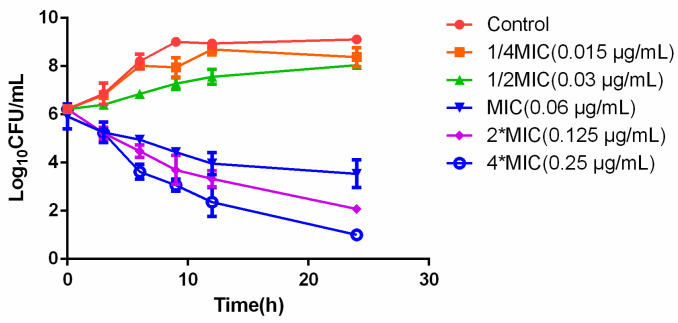
The in vitro time-killing curve of CEF against HB13 in MHB.

**Figure 2:**
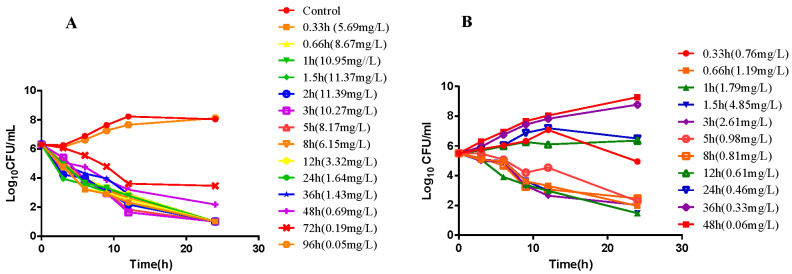
The ex vivo killing curve of CEF against HB13 in plasma (**A**) and BALF from infected pigs (**B**).

In vitro time-killing curves of ceftiofur against HB13 are shown in Figure 1. For the control, bacteria exposed to no drug and grow exponentially until approaching a maximum bacterial concentration. When *P. multocida* exposed to a higher drug concentration, a biphasic killing curve is presenting which indicated for a decline in the killing rate. Ex vivo time-killing curves were shown as Figure 2, integrating the variations in bacterial count after 24 h exposure versus PK/PD index to establish the PK/PD model.

### 2.3. Pharmacokinetic of Ceftiofur in Infected Pigs

**Figure 3:**
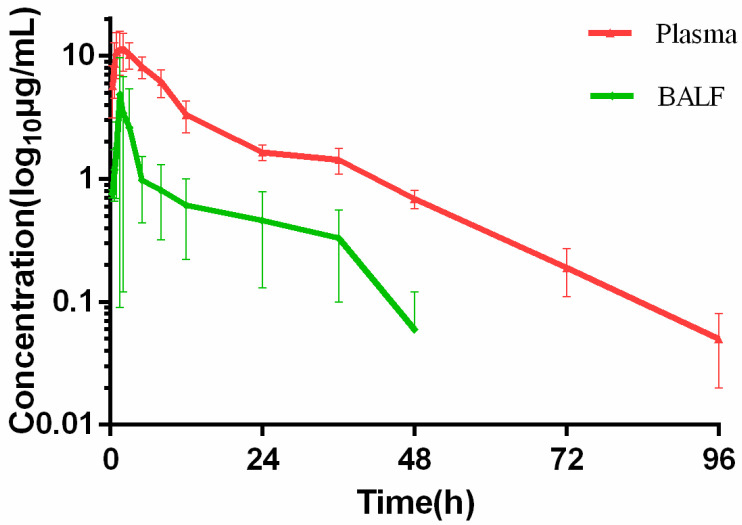
Time-concentration of DFC in plasma and BALF at different time points.

The concentration-time of DFC in plasma and BALF were shown as Figure 3. In addition, drug concentrations of BALF have been corrected by the urea ratios of plasma: BALF. The results of dilution ratio for BALF were shown in Table S2. The PK parameters of plasma and BALF in infected pigs were shown as Table 2 which were calculated by non-compartmental analysis using WinNonlin software. The maximum concentration of DFC in plasma (11.81 µg/mL) is approximately twice as in BALF (5.05 µg/mL). AUC_0–∞_ in plasma (163.04 µg·h/mL) is higher than in BALF (31.45 µg·h/mL) which indicates ceftiofur distribution is hindered into interstitial fluid of lung.

### 2.4. PK/PD Model and Dosing Regimen

By integration with in vivo PK and the ex vivo PD, the PK/PD indices can be determined both in plasma and BALF. The PK/PD indices, AUC_24 h_/MIC, and bacterial variation were well correlated by the inhibitory sigmoid E_max_ model. The model parameters of the different antibacterial effect were presented in the Table 3.

We integrated the previous study with our results to derive a MIC distribution of ceftiofur against 251 isolates of *P. multocida*, and MIC_90_ = 0.06 µg/mL was used to calculate the dose [7]. By Monte Carlo simulation, for 90% target attainment rate, the dosage was determined as 0.22 mg/kg for bacteriostatic effect (E = 0), 0.46 mg/kg for bactericidal effect (E = −3) and 0.64 mg/kg for elimination effect (E = −4), respectively. The results of Monte Carlo simulation can be found in the Supplementary Materials.

### 2.5. Validation of Dosage Regimen

**Figure 4:**
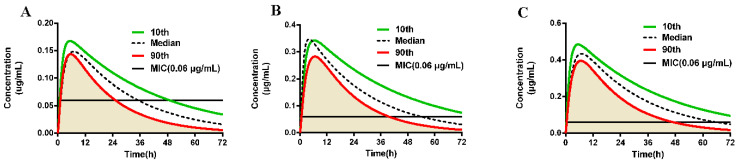
Monte Carlo analysis of free DCF concentrations in plasma for different doses using the population PBPK model. (**A**) represents 0.22 mg/kg; (**B**) represents 0.46 mg/kg; (**C**) represents 0.64 mg/kg.

The values and distributions of sensitive parameters used in the Monte Carlo analysis were shown in Table 4. The PBPK model was integrated with Monte Carlo analysis to evaluate the dosage regimen. The results of Monte Carlo analysis are presented in Figure 4 and the AUC_24 h_/MIC of 90 percentiles population are simulated as 45.03, 86.17, and 119.50 h for different antibacterial effect, respectively. The AUC_72 h_/MIC are simulated as 66.10, 123.50, and 177.33 h for different antibacterial effect, respectively. In a word, the doses, which are derived from the ex vivo PK/PD model, can achieve 90% attainment for different antibacterial effect.

**Figure 5:**
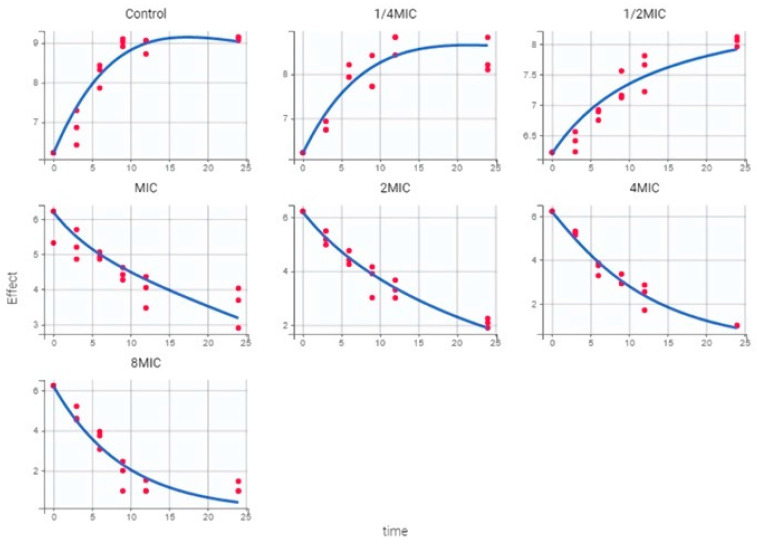
Model-prediction and observed of CEF against *P. multocida* over 24 h from the in vitro time-killing curves, i.e., observed bacterial concentration log_10_CFU/mL, IPRED: individual model predicted natural logarithm of bacterial concentration (log_10_CFU/mL).

Parameter estimates from the semi-mechanistic PD model (bacterium-specific parameters and drug-specific parameters) are summarized in Table 5. The maximal bacterial killing rate (E_max_) of CEF was 0.11 1/h, yielding a 0.6-fold increase in death rate. To achieve half of the maximal killing effect (*EC_50_*), a CEF concentration of 0.14 mg/L was required, is 2.3-fold of MIC (MIC = 0.06 µg/mL). The plot of the observed bacterial counts (log_10_ CFU/mL) versus time is presented in Figure 5. The core set of diagnostic graphs for semi-mechanistic PD model can be found in the Supplementary Materials (Figures S2–S4).

**Figure 6:**
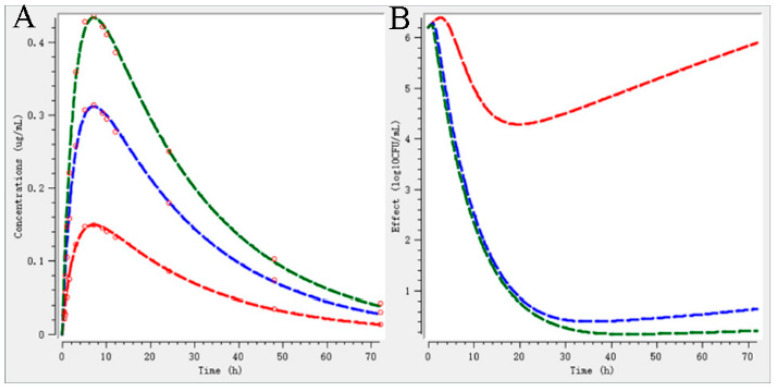
The prediction of bacterial growth kinetics under different doses. (**A**) represents the time course of drug concentrations; (**B**) represents the bacterial count change under drug exposure. (Red: 0.22; blue: 0.46; green: 0.64 mg/kg). Note: Red squares are the drug concentration for different doses by the simulation of PBPK model.

The PK data for different doses were obtained from a validated PBPK model proposed by Lin et al. [19]. The free faction of in plasma can be found in Figure S1. The PK parameters are calculated by WinNonlin, as Table S2 shown. Integrating PBPK model and semi-mechanistic PD model, the *P. multocida* responses to various exposures over 72 h were predicted as Figure 6 shown. As the bacteriostatic effect, CEF can initially inhibit the growth of *P. multocida* and the bacteria begin to regrow at 24 h after drug administration which means an additional injection by 0.22 mg/kg is necessary. For the bacterial effect, CEF can perform the antibacterial effect about 4-log10 CFU. After 72 h exposure, the bacterial has been reduced to 1-log_10_ CFU. In a conclusion, the doses for bacterial effect and elimination effect derived from the ex vivo PK/PD model can well perform the antibacterial effect which are evaluated by PBPK/PD model.

## 3. Discussion

*P. multocida* is frequently associated with the outbreak of swine respiratory tract disease. Ceftiofur is effective to cure the disease infected by *P. multocida*. It is essential to determine an optimal dosage regimen to guide the clinical medicine and protect the effect of ceftiofur.

Antibacterial resistance is a major public health issue. Due to irrationally use veterinary antibiotics, the selective pressure led to the emergence and raised antibacterial resistance in animals and humans. Generally, *P. multocida* is susceptible to the majority antimicrobials; however, the emergence of multidrug-resistant pathogenic bacteria has been widely reported in recent [20]. This may cause the cure rate declined and that even threaten human health. Optimizing and improving the dosage regimen of existing antibiotics are the available method to alleviate the resistance and ensure the antibacterial effect. PK/PD model, a recognized tool to determine the dosage regimen, has been applied in veterinary for the determination of dosage regimen [10].

The determination of dosing regimens for sick pigs is primarily based on PK data of drugs in healthy pigs. Different physiological conditions between healthy and infected animals may change PK characteristics. It is important to evaluate dosage for infected animals. Compared with the previous study, the PK parameters (AUC_0-∞_, C_max_, and T_1/2_) of plasma between healthy and *P. multocida*-infected groups were significant (*p* < 0.01) [21]. C_max_ and AUC_0–∞_ of the infected pigs decreased by 47% and 56%, respectively, compared with the healthy pigs. The T_1/2_ of the infected pigs and the healthy pigs was 13.28 and 19.51 h, respectively. PK profile can be altered in the respiratory tract of infected swine which has been found in the published [22]. The reason may be the verities from physiological and biochemical indices, such as body temperature, renal clearance, etc. *P. multocida*-infected leading to significant changes of some PK parameters compared with the healthy has also been reported by the published [23,24]. As Li’s results, all PK parameters of ceftiofur between the *Haemophilus parasuis*-infected and healthy swine, show no significant differences [25]. This possibly because the different degrees of respiratory tract disease.

PELF is an important site of infection in pneumonia, different sampling methods have been employed to collect PELF such as microdialysis, bronchial swabs, and bronchoalveolar lavage (BAL). We have applied BAL to gain the interstitial fluid of lung in infected group. The PELF AUC is 25% of Plasma AUC. It is important to evaluate a dose after administration that is sufficient to kill the microorganism in the target tissue. As Figure 3 showed, DFC concentration in BALF is higher than 0.06 μg/mL at the last time point (48 h), which indicated that ceftiofur can maintain an active concentration in pulmonary of infected pigs.

Bacterial reduction after 24 h incubated and PK/PD index are fitted into the sigmoid E_max_ model to derive values of PK/PD index for bacteriostatic, bactericidal, and bacterial elimination. PK/PD indices play important roles to determine the dosages. As the beta-lactam with a longer half-life, AUC/MIC has been approved to represent the antibacterial effect of CEF [26]. The ex vivo model has applied AUC/MIC as the PK/PD index to evaluate the dosage regimen of CEF. The PK/PD indices of CEF against *H. parasuis* is 36.0, 71.6, and 90.6 h, respectively for the bacteriostatic, bactericidal, and bacteriophagous action [25]. And, for *A. pleuropneumoniae,* the PK/PD indices were determined as 45.7, 63.8, and 69.0 h for the different actions [21]. Due to the difference of the respiratory tract pathogens to CEF, it is essential to determine the PK/PD index for different pathogens.

PBPK model is a mechanism-based approach to simulate the absorption, distribution, metabolism, and elimination of chemicals, which is characterized as extrapolating to different therapeutic scenarios and species [27]. A PBPK model of CEF and its main metabolism (DCF) in swine has been established by Lin et al. [19]. We applied this PBPK model to predict the concentration of free fraction drug in plasma for different doses. In addition, as Lin introduced, by changing the value of hepatic metabolism rate, the PK profile of CEF in sick status can be simulated. This corresponds to the downregulation of hepatic drug-metabolizing enzymes, such as 40~85% decrease of total CYP450 activity in sick status [28]. Sensitive parameters, including hepatic metabolism rate, corresponding to various distributions are inserted into Monte Carlo analysis to perform a population PBPK model. In addition, combined with PK/PD models, the efficacy of dose can be predicted [29]. Zhou has determined the dosage regimen of enrofloxacin against pathogens in the intestinal tract with the PBPK model and PK/PD parameters [30]. As Figure 4 shown, at least 90% population can reach the PK/PD parameters for different doses. This is a novel method to evaluate PTA which contributes to the assessment of dosage regimen.

Semi-mechanistic PD model can integrate with different types of PK model, such as population PK model and PBPK model, to evaluate the dosage. A whole-body PBPK model has been integrated with semi-mechanistic PD model to establish a PBPK/PD model to evaluate the potency of antibiotic in different tissues [31]. In this manuscript, the semi-mechanistic PD model is proposed by Nilsen, a resting subpopulation introduced into the model to describe bacterial growth phase and antibacterial action [32]. This model has been adapted to evaluate the PK/PD indices of florfenicol against *P. multocida* [17]. It has also been applied to evaluate the dosage regimen of tylosin against *Staphylococcus delphini* [18]. In addition, semi-mechanistic PD model can characterize the emergency of resistance, which will provide an available way to identify dose regimens that can prevent bacterial drug resistance [33].

There are some limitations in the present manuscript. For the mathematical models, much more data are needed that can improve the predictive ability and that is also helpful to investigate PK or PD mechanism. More *P. multocida* strains should be selected for the PD experiments. However, by mathematical model, the varieties among individuals can be involved. Our manuscript is determined the dose of CEF against *P. multocida* can be calculated as 0.22, 0.46, and 0.64 mg/kg for static, 99.9% killing and 99.99% killing, respectively. The recommended dosage regimen of ceftiofur for the treatment of swine respiratory disease is 3–5 mg/kg body weight administered intramuscularly once daily for 3–5 consecutive days. The reason for the difference from the recommended dose may due to the high susceptivity of *P. multocida* to CEF (MIC_90_ is 0.06 ug/mL). The assessments of PBPK model and PBPK/PD model indicate that 0.46 mg/kg body weight of ceftiofur can achieve the therapeutic effect to *P. multocida*.

## 4. Materials and Methods

### 4.1. Animals

Six healthy crossbred pigs having 20 ± 2 kg average body weight. Prior to the experiments, all pigs were raised 7 days to acclimate. All the animal experiments were approved by Institutional Animal Care and Use Committee at Huazhong Agricultural University (approval number HZAUSW-2018-021) and Technology Agency and performed according to the committee guidelines. After animal experiments, all animals were euthanized.

### 4.2. Bacteria and Antibiotic

*P. multocida* strains (HB13), stored in −80 °C by milk, were determined minimum inhibitory concentration (MIC). Prior to MIC determination, it was identified by PCR for the positive [34]. Tryptone soya agar (TSA) and Mueller–Hinton broth (MHB) were used to culture *P. multocida*. Ceftiofur standard (China Institute of Veterinary Drug Control, 89.5%) and desfuroyl ceftiofur standard (Toronto Research Chemicals, 90%) were used in PK/PD modeling. Ceftiofur hydrochloride injection was purchased from Shanghai full woo Biotechnology Co., Ltd. (Zhumadian, China).

### 4.3. Pharmacodynamics

#### 4.3.1. Determination of MIC

HB13 was determined with serial two-fold dilution by broth microdilution technique following the guidelines of the CLSI, at concentration between 8 and 0.0015 μg/mL. The MIC contained a minimum amount of ceftiofur where the visible growth of bacteria was inhibited.

#### 4.3.2. In Vitro and Ex Vivo Pharmacodynamic of CEF against *P. multocida*

Minimum bactericidal concentration (MBC) was the minimum concentration of CEF inhibiting 99.9% bacterial density. For the minimum bactericidal concentration (MBC), the 100-µL suspension from the 96 well plates of CEF were diluted with MHB by 1:10 steps and 100-µL were spread on TSA agar plates for colony forming unit (CFU) counting and incubated after at 37 °C with 5% CO_2_ for 24 h. MIC and MBC of CEF against HB13 are also performed in plasma, respectively.

The mutant prevention concentration (MPC) of ceftiofur was determined by agar method. *P. multocida*, which cultured to 10^10^ CFU/mL, was respectively inoculated onto the plates with MIC, 2 × MIC, 4 × MIC, 8 × MIC, and 16 × MIC, and then incubated at 37 °C in an atmosphere containing 5% CO_2_ for 96 h. Linearly decreasing of cefquinome concentration by 20% for the plates without bacteria until the minimum concentration of drug with no bacterial growth.

The post-antibiotic effect (PAE) described the continuous inhibition of bacterial growth by removal the antibiotic exposure. HB13 was co-incubated for 1 or 2 h with MIC, 2 × MIC, and 4 × MIC of ceftiofur. The recovery growth kinetic curves of bacteria were established in order to calculate the PAE. PAE = T − C, where T is the time for the growth of 1log_10_ CFU for drug exposure with different time culture, C is the time for the growth of 1log_10_ CFU for blank plates.

For ex vivo time-killing curves, the bacteria (about 5 × 10^6^ CFU/mL) were cultured on the plasma and BALF, respectively, obtained from pigs at different time points after drug administration. The viable counts of bacteria are determined at 0, 3, 6, 9, 12, and 24 h. At each time point, 100-µL aliquots were serially diluted by saline and then colony forming units for counting after 24 h of incubation. The limit of detection was 10 CFU/mL.

### 4.4. Pharmacokinetics

Six pigs were inoculated *P. multocida* (HB13), in the logarithmic growth phase, with 3~5 mL into each nostril about 10^6^ CFU/mL on two consecutive days for four times. Fever, cough, and dyspnea were found as symptoms of *P. multocida* infected. Each pig was intramuscularly (i.m.) administrated ceftiofur hydrochloride injection on the neck at a single dose of 5 mg/kg bodyweight (BW).

Blood samples were collected at 0.33, 0.66, 1, 1.5, 2, 3, 5, 8, 12, 24, 48, 72, and 96 h after drug administration. Bronchoalveolar lavage fluid (BALF) samples were collected at 0.33, 0.66, 1, 1.5, 2, 3, 5, 8, 12, 24, and 48 h. Then, blood samples were centrifuged at 3500× *g* and for 10 min and BALF samples were centrifuged at 800× *g* for 10 min. The supernatants were stored in −20 °C prior to HPLC and ex vivo pharmacodynamic experiment.

During the collection of BALF, it is essential to carry out an anesthesia scheme for pigs. Briefly, 30 min before anesthesia, pigs were intramuscularly administered atropine (0.05 mg/kg) and propofol (5 mg/kg) was slowly injected into pigs by the intravenous administration. According to the standard procedures reported by Lee [35], an electronic fiberoptic bronchoscope (Kangmei GU-180VET) was used to obtain the BALF. The electronic fiberoptic bronchoscope was slowly inserted into the right lung lobe of pigs when pigs were under anesthesia. Then, 50 mL of warm saline was instilled into the lung lobe with a syringe, and the BALF sample was collected in a 50-mL centrifuge tube after waiting 20 s.

### 4.5. HPLC Analysis

Ceftiofur is rapidly metabolized to desfuroylceftiofur (DFC) in animals after administration. The concentration of DFC represents ceftiofur concentration in samples. And the HPLC method to measure DFC concentration is developed by Li et al. [25].

Sample preparation: 0.5 mL of plasma or BALF samples were added into 7 mL of DTE-borate buffer. The samples were incubation at 50 °C for 15 min, with a 30 s vortexing for every 5 min, and then centrifuged at 3500 r/min for 10 min. An Agilent HLB column (60 mg/3 cc) was used as solid extraction which was activated and equilibrated consecutively with 3 mL of methanol and ultrapure water. Materials, after centrifuging, were added to the HLB column and a flow rate set at 1 mL/min. Eluent was concentrated with nitrogen and diluted to 0.5 mL by ultrapure water. Finally, filtered through the 0.22-μm filter and prepared for HPLC.

HPLC was performed by using a Waters 2695 series HPLC and a Waters 2587 UV detector set at a wavelength of 266 nm which was equipped with Agilent SB-aq (280 × 4.6 mm i.d., 5 µm; Agilent Technology, Santa Clara, CA, USA). The injection volume was 50 µL and the temperature should be maintained at 30 °C. The mobile phase consisted of A (0.1% trifluoroacetic acid) and B (acetonitrile) with isocratic elution as follows (A:B = 86:14 (*v*/*v*) at 1 mL/min flow rate.

The limit of determination (LOD) of DFC was 0.02 μg/mL and limit of quantification (LOQ) of DFC was 0.1 μg/mL both in plasma and BALF. DFC quantification was linear within a range of 0.01–20 μg/mL (y = 62,037x − 2449.9, r = 0.9996). The working curves of DFC in plasma (y = 43,507x + 9311.2, r = 0.9979) and in BALF (y = 49,991x + 5743.1, r = 0.9994). The inter-day and intra-day coefficient variation were <5%. Moreover, the recovery ratios values were in the range of (78–89%) in plasma and (83–88%) in BALF for DFC detection.

For collection of BALF, there is an error between the volumes of injection and extraction. The urea dilution method was performed to correct the concentration of diluted BALF [36]. Briefly, the BUN kit (Ningbo Ruiyuan Biotechnology Co., Ltd, Ningbo, China.) was used to determine the concentration of urea in plasma and BALF by a Microplate Reader (μQuant, BioTek, Winooski, VT, USA). The PK data of plasma and BALF was analyzed by WinNonlin V5.2.

### 4.6. PK/PD Model

The PK/PD index (AUC/MIC or T > MIC) correlating net reduction of bacterial count at 24 h from an initial inoculum count was selected by fitting the sigmoid E_max_ model.

$$E=E_{0}-\frac{I_{\max}\cdot {\mathrm{INDEX}}^{N}}{{\mathrm{INDEX}}^{N}+{\mathrm{INDEX}}_{50}{}^{N}}$$

where E_0_ was the change in log_10_ CFU/mL after 24 h incubation in the control sample compared with the initial incubation. The maximum possible observed effect is I_max_. INDEX was the value of PK/PD index (AUC_24 h_/MIC or T > MIC). INDEX_50_ was the value of AUC_24 h_/MIC or T > MIC producing a 50% reduction in bacterial counts from initial inoculum, and N was the Hill coefficient that described the steepness of the curve.

For different antibacterial effect as bacteriostatic (E = 0, no change from initial counts), bactericidal (E = −3, 3-log_10_ reduction from initial count) and bacterial elimination (E = −4, 4-log_10_ reduction from initial count), the values of best PK/PD index were calculated.

### 4.7. Dose Determination

The PK/PD index was substituted into the following equations to calculate dosage regimen for different antibacterial effect:

$$\mathrm{Dose}=\frac{({\mathrm{AUC}}_{24\;h}/\mathrm{MIC})\times {\mathrm{MIC}}_{90}\times \mathrm{CL}}{\mathrm{fu}\times F}$$

where AUC_24 h_/MIC was the value of PK/PD index in plasma for the different effect (bacteriostatic, bactericidal, and bacterial elimination), CL was the clearance, fu was the fraction of drug in plasma (42%) [19] and F was bioavailability. In addition, the populational dosages, achieving 90% target attainment rate, were conducted for 10,000 trails by Monte Carlo simulation (Crystal Ball software, version 7.2.2) [37].

### 4.8. Evaluation of Dosage Regimen

The physiological based pharmacokinetic (PBPK) model of ceftiofur and its metabolism (DCF) in swine has been established by Lin et al. [19], whereas the drug concentrations in lung compartment were not validated. The PK profiles only in the plasma were predicted.

#### 4.8.1. PBPK Model

The doses of CEF against *P. multocida* for different antibacterial effect as bacteriostatic, bactericidal and elimination were calculated from ex vivo PK/PD model. Input the doses into the PBPK model, respectively, and the concentrations of unbound drug for different effects can be determined.

Monte Carlo analysis can integrate with PBPK model to estimate the effects of parameter uncertainties and the intra-species variability of experimental animals which will firstly be used to evaluate the dosage regimen. Based on the previous population PBPK model, the sensitive parameters, which are presented in the previous [19], were assumed as normal or log-normal distribution with different coefficients of variance within a 95% confident interval as Table 4 shown.

By Monte Carlo analysis for 1000 iterations, the area under concentration (AUC) of unbound fraction for different dosages was calculated. Compared with the PK/PD parameters, the doses will be assessed to achieving 90% target attainment rate or not. The PBPK model was constructed in Berkeley Madonna Version 8.3.23.0.

#### 4.8.2. Semi-Mechanistic PD Model

For in vitro time-killing curves, the bacteria (10^6^ CFU/mL) were cultured with two-fold dilution of CEF ranging from 1/4 to 8 × MIC. Growth was checked with a control. The tubes were incubated at 37 °C with 5% CO_2_ and the viable counts of bacteria were determined at 0, 3, 6, 9, 12, and 24 h. At each time point, 100 µL of aliquots were serially diluted by saline and then colony forming units for counting after 24 h of incubation. The limit of detection was 10 CFU/mL. Each time-kill experiment was carried out in triplicate on separate occasions.

**Figure 7:**
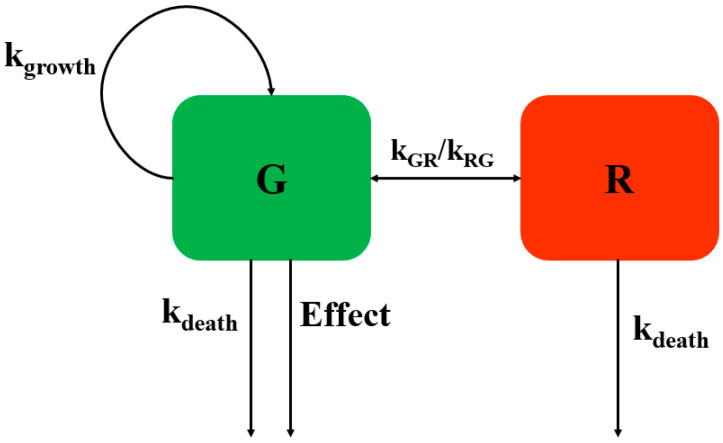
Schematic illustration of the semi-mechanistic PD model. The PD model includes one proliferating subpopulation (G) and one resting subpopulation (R). The bacterial system is described with first-order rate constants for multiplication of the bacteria in the growing subpopulation (k_growth_), for the degradation of bacteria in both subpopulations (k_death_), and for the transfer between the compartments (k_GR_ and k_RG_). The antibiotic concentration in the BioPhase compartment is assumed to stimulate the killing rate of bacteria in the susceptible stage according to an E_max_ model (Effect).

As Figure 7 shown, two bacterial subpopulations are adapted to describe the data of in vitro time killing curves. In addition, for the two bacterial subpopulations, susceptible growing subpopulation and resting subpopulation are described by bacterium-specific parameters (k_growth_, k_death_, k_GR_) and drug-specific parameters (E_max_, EC_50_, γ). Finally, the microbial growth, under various free drug exposures, was predicted over 72 h.

The bacterial growth in G is assumed to be regulated by the natural growth rate, the natural death rate and the kill rate of an antimicrobial drug.

$$\frac{\mathrm{dG}}{\mathrm{dt}}=k_{\mathrm{growth}}\times G-\mathrm{EFFECT}\times G-k_{\mathrm{death}}\times G-k_{\mathrm{GR}}\times G$$

where G (CFU/mL) is bacterial concentration in the G compartment, t (h) is time, and k_growth_, k_death_, and EFFECT are rate constants of bacterial growth, bacterial natural death, and bacterial kill by CEF, respectively. k_GR_ (1/h) is a rate constant describing the rate of transfer from the G to the R subpopulation.

The bacterial growth in R is assumed to be regulated by the transformation rate and the death rate.

$$\frac{\mathrm{dR}}{\mathrm{dt}}=k_{\mathrm{GR}}\times G-k_{\mathrm{death}}\times R$$

where R (CFU/mL) is bacterial concentration in the R compartment, t (h) is time.

The transfer rate (k_GR_) is assumed to be regulate by bacterial growth and bacterial count in the system.

$$k_{\mathrm{GR}}=\left(k_{\mathrm{growth}}-k_{\mathrm{death}}\right)*\frac{\left(G+R\right)}{B_{\max}}$$

The effect of CEF is assumed to follow a non-linear function that depends on the concentration in the system and is described by an E_max_ sigmoid model:

$$\mathrm{EFFECT}=\frac{E_{\max}\times C^{\gamma }}{{\mathrm{EC}}_{50}^{\gamma }+C^{\gamma }}$$

where E_max_ (1/h) is maximum bacterial kill by CEF representing drug efficacy, EC_50_ (mg/L) is the concentration of CEF that produces half of the maximum effect measuring drug potency, gamma (γ-scalar) is a sigmoidicity coefficient expressing the slope of antimicrobial effect curves and presenting drug sensitivity, and C (mg/L) is the concentration of CEF at time (t).

The software Monolix 2018 R1 (Lixoft, Antony, France) was used to perform all parameter estimations. The parameters in the model were estimated using stochastic approximation expectation maximization (SAEM) over a maximum of 200 iterations with a Monte Carlo sampling size of 10,000. To discriminate between nested models, the difference in the objective function value (−2 log likelihood) was used. A decrease in OFV of 3.84 between nested models with one parameter difference was a statistically significant difference at the 5% significance level.

Export the concentrations for different time points which were predicted by PBPK model, PK parameters are calculated for classical compartments model by WinNonlin version 5.2.1. In addition, mathematical equations of PK and semi-mechanistic PD model were descripted in Mlxplore. The parameters were also inputted. The PK profiles can be simulated by Mlxplore, simultaneously, the response of *P. multocida* can be predicted. And the efficacy of dose can be evaluated.

## Figures and Tables

**Table 1:** Post antibiotic effect (PAE) of CEF against *P. multocida* after 1 h and 2 h.

Ceftiofur (µg/Ml)	PAE (h)	
	1 h Exposure	2 h Exposure
1 × MIC	0.28	0.33
2 × MIC	0.43	0.62
4 × MIC	0.60	1.01

**Table 2:** The pharmacokinetic parameters of CEF in infected pigs by I.M. at 5 mg/kg·bw.

Parameter	Units	Plasma (*n* = 6)	BALF (*n* = 4)
T_max_	h	1.92 ± 0.68	1.38 ± 0.18
C_max_	µg/mL	11.81 ± 3.09	5.05 ± 3.22
AUC_0-t_	µg·h/mL	159.05 ± 15.98	30.31 ± 16.01
AUC_0-∞_	µg·h/mL	163.04 ± 15.57	31.45 ± 16.79
CL/F	L/kg/h	0.03 ± 0.00	0.24 ± 0.10
Vd/F	L/kg	0.60 ± 0.16	3.97 ± 1.99
MRT	h	15.88 ± 1.36	13.85 ± 0.55
Ke	h^−1^	0.06 ± 0.01	0.06 ± 0.01
T_1/2_	h	13.28 ± 3.23	11.59 ± 1.97

Note: T_max_: peak time; C_max_: maximum concentration; AUC: the area under the concentration-time curve; CL/F: clearance rate corrected by bioavailability; Vd/F: apparent distribution volume corrected by bioavailability; MRT: mean residence time; Ke: elimination rate constant; T_1/2_: elimination half-life.

**Table 3:** Integration PK/PD model of CEF against *P. multacia* in plasma of infected pigs.

Parameters	Unit	Plasma	BALF
I_max_	Log_10_ CFU/mL	9.78 ± 0.37	7.41 ± 0.12
E_0_	Log_10_ CFU/mL	3.57 ± 0.43	3.59 ± 0.11
IC_50_	h	60.14 ± 3.48	59.84 ± 0.52
N	-	1.94 ± 0.55	4.10 ± 0.08
AUC_24 h_/MIC (E = 0)	h	44.02 ± 3.17	58.99 ± 4.82
AUC_24 h_/MIC (E = −3)	h	89.40 ± 4.48	99.69 ± 2.81
AUC_24 h_/MIC (E = −4)	h	119.90 ± 19.75	-

**Table 4:** Values and distributions of parameters used in the Monte Carlo analysis for the PBPK model.

Abbreviation	Distribution	Mean	SD	CV	Upper Bound	Lower Bound
QCC	Normal	5	1.5	0.3	7.940	2.060
QKC	Normal	0.12	0.036	0.3	0.191	0.049
BW	Normal	40	12	0.3	63.520	16.480
VLC	Normal	0.0247	0.00741	0.3	0.039	0.010
PL	Lognormal	0.13	0.052	0.4	0.002	0.810
PK	Lognormal	0.4	0.16	0.4	0.055	1.446
PM	Lognormal	0.06	0.024	0.4	0.0002	0.410
PL1	Lognormal	0.13	0.052	0.4	0.002	0.810
PK1	Lognormal	0.4	0.16	0.4	0.055	1.446
PM1	Lognormal	0.06	0.024	0.4	0.107	0.013
KmC	Normal	1	0.3	0.3	1.588	0.412
Frac	Normal	0.7	0.21	0.3	1.112	0.288
Kurine1C	Normal	0.01	0.003	0.3	0.016	0.004

Note: The parameters were the sensitive parameters derived from Lin’s study. QCC: cardiac output; QKC: kidney blood flow (fraction of cardiac output); BW: bodyweight; VLC: Liver volume (fraction of bodyweight); PL, PK, PM: liver, kidney, and muscle: plasma partition coefficient of the parent drug; PL1, PK1, PM1: liver, kidney, and muscle: plasma partition coefficient of the metabolite; KMC: hepatic metabolic rate; Frac: fraction of parent drug metabolized to the main metabolite; Kurine1C: urinary elimination rate constant of the metabolite.

**Table 5:** Estimation of PD parameters obtained from a model of the time-killing experiment of CEF against *P. multacia*.

Parameter	Units	*P. multacia*	
		Mean	SE
k_growth_	1/h	0.2	0.28
k_death_	1/h	0.179	(fixed)
B_max_	1/h	8.48	0.28
E_max_	1/h	0.11	0.027
EC_50_	mg/L	0.14	0.031
γ	*-*	8.54	-

Note: k_growth_ is the constant of growth rate; k_death_ is the constant of death rate; B_max_ is the maximum bacterial concentration in the system. E_max_ is maximum drug effect; γ is the Hill coefficient; EC_50_ is the concentration of drug that produces half of the maximal effect.

## Supplementary Materials

The following supporting information can be downloaded at: https://www.mdpi.com/article/10.3390/ijms23073722/s1.
