# Supplementary material for: Optimization and Validation of Dosage Regimen for Ceftiofur against Pasteurella multocida in Swine by Physiological Based Pharmacokinetic–Pharmacodynamic Model
Source: Int J Mol Sci. 2022 Mar 28;23(7):3722. doi: 10.3390/ijms23073722 (PMC8998519; doi:10.3390/ijms23073722)
Supplement: Supplementary file 1 [file ijms-23-03722-s001.zip › ijms-1624214-supplementary.pdf]

Optimization and Validation of dosage regimen for Ceftiofur against *Pasteurella multocida* in swine by Physiological based Pharmacokinetic-Pharmacodynamic model

Kun Mi[1] , Shanju Pu[2], YiXuan Hou[1] , Lei Sun[1] , Kaixiang Zhou[2], Wenjin Ma[1,2], Xiangyue Xu[2], Meixia Huo[1], Zhenli Liu[1,2], Changqing Xie[1,2]， Wei Qu[1,2] and Lingli Huang[1,2 *]*

1 National Reference Laboratory of Veterinary Drug Residues (HZAU) and MAO Key Laboratory for Detection of Veterinary Drug Residues, Huazhong Agricultural University, Wuhan, Hubei 430070, China;

2 MOA Laboratory for Risk Assessment of Quality and Safety of Livestock and Poultry Products, Huazhong Agricultural University, Wuhan, Hubei 430070, China;

* **Correspondence: qw@mail.hzau.edu.cn and huanglingli@mail.hzau.edu.cn.**

Table S1 The dilution multiple of BALF in diseased pigs

Time（h）	1#	2#	3#	4#
0.33	16.55	31.62	14.76	15.52
0.66	17.62	11.46	11.33	16.86
1	7.65	13.35	4.29	11.35
1.5	15.28	43.31	16.58	20.72
2	20.95	20.58	7.79	9.82
3	13.75	18.87	10.84	9.97
5	6.99	15.9	5.89	14.47
8	10.35	6.54	12.64	16.06
12	14.5	12.64	23.01	8.87
24	9.78	6.57	16.67	11.58
36	11.46	7.92	13.78	14.77
48	9.85	14.22	17.97	12.02

Table S2 The PK parameters of CEF in plasma for compartment model

Dose (mg/kg)	Parameter	unit	Value
0.22 (Bacteriostatic action)	V/F	ml/kg	1114.11
	ka	1/h	0.3381
	k	1/h	0.0393
0.46 (Bactericidal action)	V/F	ml/kg	1114.27
	ka	1/h	0.3381
	k	1/h	0.0393
0.64 (Elimination)	V/F	ml/kg	1114.21
	ka	1/h	0.3381
	k	1/h	0.0393

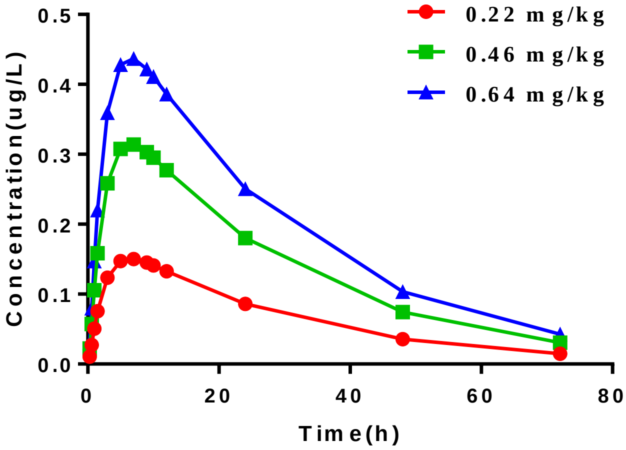

Figure S1 Plot of the simulations for concentrations in infected pigs plasma for different doses by PBPK model

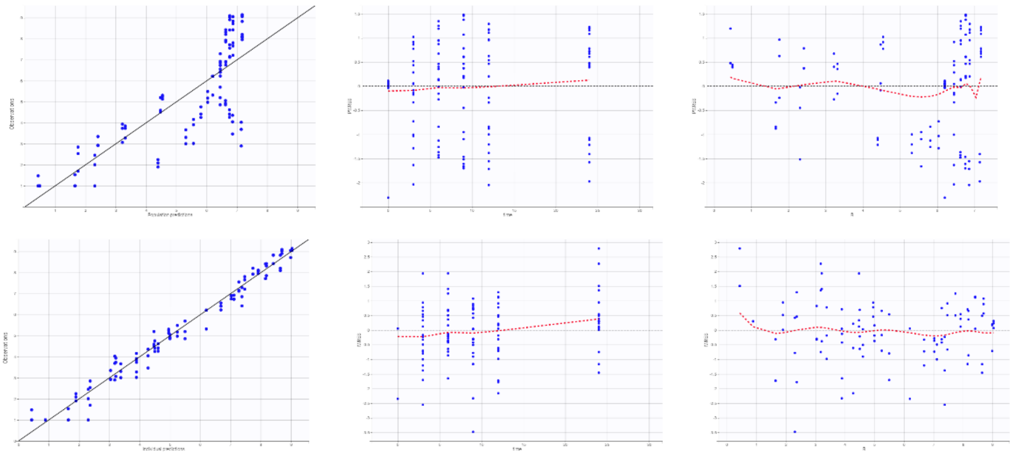

Figure S2 Basic goodness-of-fit plots. For the first line, they are about the populational fitting. From left to right, they are plot of observation vs. population predictions using monte Carlo simulation (simulation-based population predictions (PPRED)), plot of population weighted residual (PWRES) vs. time and plot of population weighted residual (PWRES) vs. population predictions, respectively. For the second, they are about individual fitting. From left to right, plot of observation vs. population predictions individual predictions (IPRED), plot of ndividual weighted residuals (IWRES) vs. time and plot of ndividual weighted residuals (IWRES) vs. population predictions, respectively.

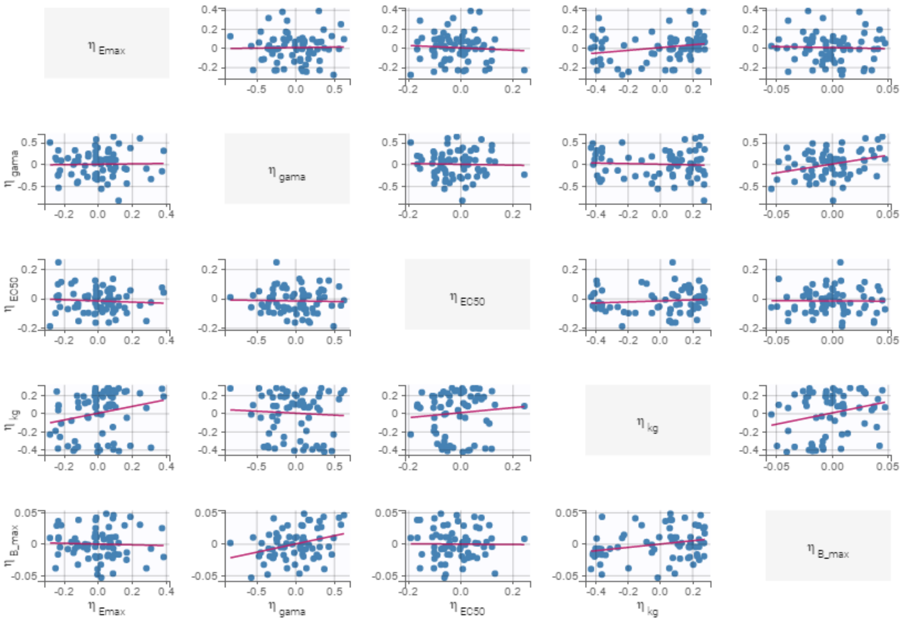

Figure S3 Plots of Correlation between random effects.

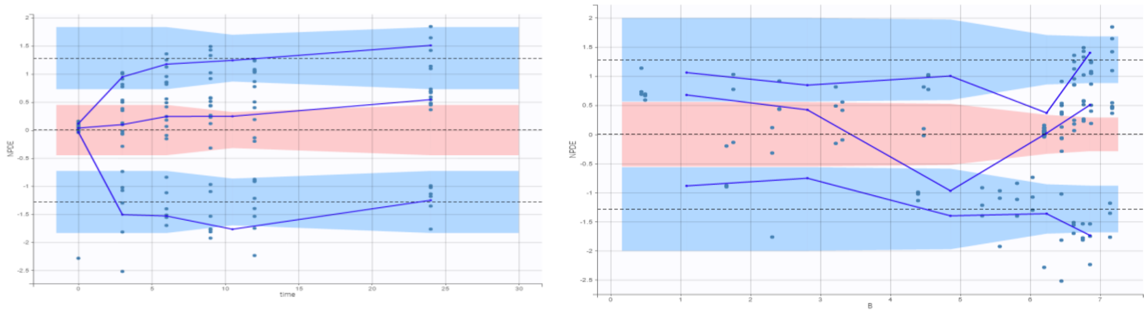

A B

Figure S4 Correlation between random effects. A represents Normalized prediction distribution error (NPDE) vs. time. B represents Normalized prediction distribution error (NPDE) vs. population predictions

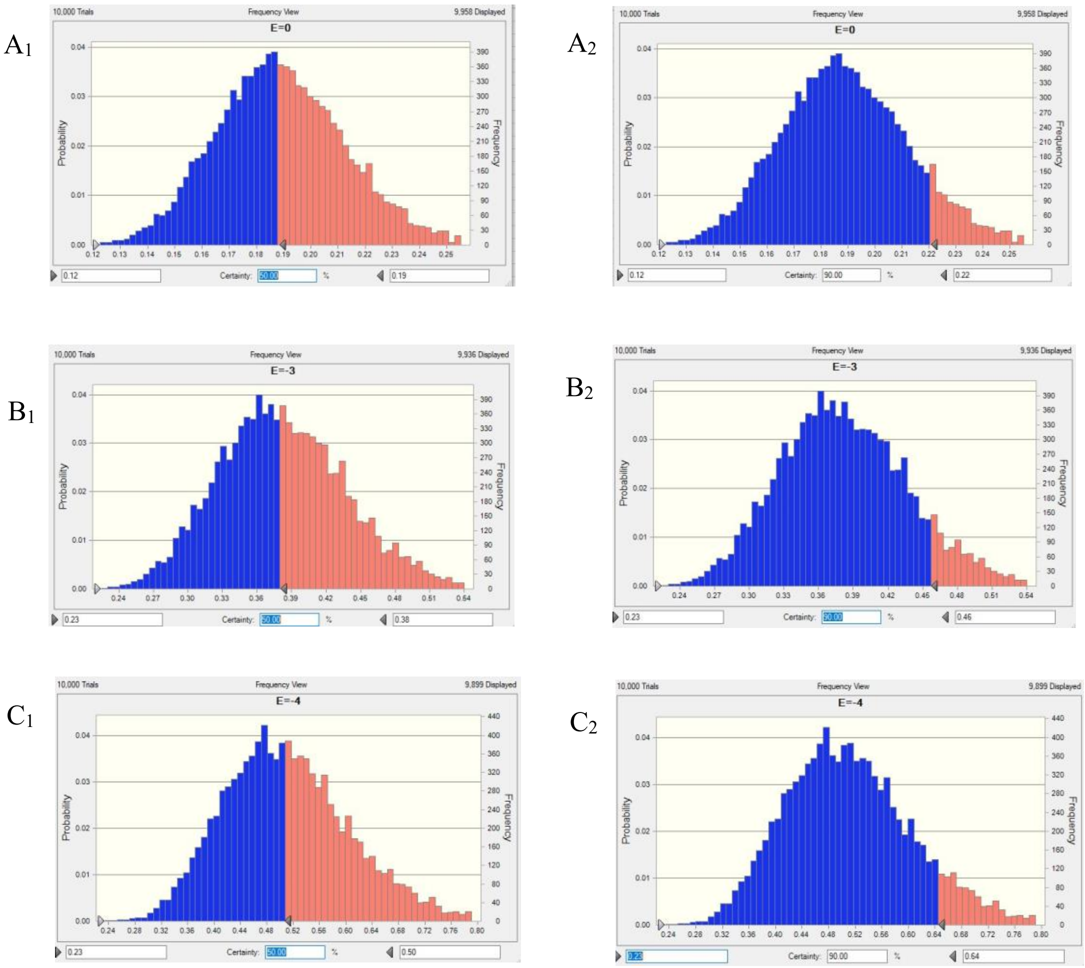

Figure S5 The predicted regimens of CEF against *P. multocida* for 50% and 90% TAR.

(A_1: 50% TAR for bacteriostatic. B_1: 50% TAR for bactericidal. C_1: 50% TAR for elimination. A_2: 90% TAR for bacteriostatic. B_2: 90% TAR for bactericidal. C_2: 90% TAR for elimination)

Equation:

1. **The effect of CEF is assumed to follow a non-linear function that depends on the concentration in the system and is described by an E_{max} sigmoid model**

$$EFFECT = \frac{E_{max} \times C^{\gamma}}{EC_{50}^{\gamma} + C^{\gamma}}$$

where E_{max} (1/h) is maximum bacterial kill by CEF representing drug efficacy, EC_{50} (mg/L) is the concentration of CEF that produces half of the maximum effect measuring drug potency, gamma (γ-scalar) is a sigmoidicity coefficient expressing the slope of antimicrobial effect curves and presenting drug sensitivity, and C is the concentration of CEF at time (t).

2. **The bacterial growth in G is assumed to be regulated by the natural growth rate, the natural death rate and the kill rate of an antimicrobial drug.**

$$\frac{dG}{dt} = k_{growth} \times G - EFFECT \times G - k_{death} \times G - k_{GR} \times G$$

where G (CFU/mL) is bacterial concentration in the G compartment, t (h) is time, and K_{growth}, K_{death} and EFFECT are rate constants of bacterial growth, bacterial natural death, and bacterial kill by CEF, respectively. K_{GR} (1/h) is a rate constant describing the rate of transfer from the G to the R subpopulation.

3. **The bacterial growth in R is assumed to be regulated by the the transformation rate and the death rate.**

$$\frac{dR}{dt} = k_{GR} \times G - k_{death} \times R$$

where R (CFU/mL) is bacterial concentration in the R compartment, t (h) is time.

4. **The transfer rate (kGR) is assumed to be regulate by bacterial growth and bacterial count in the system.**

$$k_{GR} = (k_{growth} - k_{death}) * (1 - \frac{G + R}{B_{max}})$$
